# Causal relationship between genetically predicted uterine leiomyoma and cancer risk: a two-sample Mendelian randomization

**DOI:** 10.3389/fendo.2024.1429165

**Published:** 2024-08-29

**Authors:** Chenyang Zhao, Anquan Shang, Han Wu, Qiong Li, Lixiu Peng, Chaoyan Yue

**Affiliations:** ^1^ Department of Obstetrics and Gynecology, The First People’s Hospital of Chenzhou, Chenzhou, China; ^2^ Department of Laboratory Medicine, The Second People’s Hospital of Lianyungang & The Oncology Hospital of Lianyungang, Xuzhou Medical University Lianyungang Second Hospital & Jiangsu University Lianyungang Second Hospital, Lianyungang, China; ^3^ Clinical Laboratory, Shanghai Children’s Medical Center, School of Medicine, Shanghai Jiao Tong University, Shanghai, China; ^4^ Obstetrics and Gynecology Hospital of Fudan University, Shanghai, China

**Keywords:** cancer, causal association, hormone, Mendelian randomization, uterine leiomyoma

## Abstract

**Purpose:**

Studies have demonstrated that hormonal imbalance, such as elevated level of estrogen or reduced level of progesterone, was the main inducing factor of uterine leiomyoma (UL) development and some cancers. UL has been reported to be associated with several cancers in observational studies. However, the causal associations between UL and cancers remain unclear.

**Methods:**

A two-sample Mendelian randomization (MR) analysis was conducted to investigate the causal associations between UL and 16 site-specific cancers using the public databases. Four methods, namely, the inverse variance weighting (IVW), MR-Egger, weighted median, and weighted mode, were applied in our MR analysis. Sensitivity tests were also performed to evaluate the robustness of these causal associations.

**Results:**

The IVW analysis indicated that genetically predicted UL increased the risk of low malignant potential ovarian cancer [odds ratio (OR) = 1.22, 95% confidence interval (CI): 1.06–1.40, *p *= 0.004], serous ovarian cancer (OR = 1.29, 95% CI: 1.10–1.52, *p* = 0.002), invasive mucinous ovarian cancer (OR = 1.24, 95% CI: 1.08–1.44, *p* = 0.003), clear cell ovarian cancer (OR = 1.25, 95% CI: 1.03–1.51, *p* = 0.023), breast cancer (OR = 1.07, 95% CI: 1.02–1.11, *p* = 0.002), and brain tumor (OR = 1.23, 95% CI: 1.06–1.42, *p* = 0.007). Conversely, genetically predicted UL reduced the risk of gastric cancer (OR = 0.91, 95% CI: 0.85–0.98, *p* = 0.008). The causal effects were consistent in the sensitivity analysis.

**Conclusions:**

Our results demonstrated that UL exhibits a causal relationship with high risk of several cancers. We suggest reinforcing the cancer screening in UL patients to enable the early detection of cancers.

## Introduction

Uterine leiomyoma (UL), more commonly known as uterine fibroid or uterine myoma, represents the predominant benign tumor affecting women of reproductive age, with an incidence rate as high as more than 70% worldwide (1). The incidence of UL increases with age, peaking in the fourth and fifth decades of life. Despite their benign feature, UL has a high level of morbidity and displayed clinical symptoms mainly related to the fibroids’ dimensions and position. Most patients with UL show no symptoms at all, or only mild ones, and they are often diagnosed incidentally during routine gynecologic examinations. The exact causes of UL remain unknown, but several risk factors have been shown to affect the formation and development of UL, which include genetic predispositions, growth regulators, hormonal imbalances, and molecular pathways (2, 3). Studies have demonstrated that hormonal disturbances, specifically elevated estrogen level or reduced progesterone, are implicated as pivotal stimuli for UL formation, echoing similar hormonal influences observed in certain cancers (4–6).

To date, several researchers have alluded to UL being correlated with a heightened risk of ovarian, breast, lung, and meningioma cancers (7–10). Conversely, contradictory findings exist, such as research suggesting no overall association between UL history and breast cancer incidence (11). These inconsistencies can be attributed to study variability in design, follow-up duration, and confounding factor management. Observational studies face challenges in inferring causality due to confounders and reverse causation, necessitating deeper exploration into the UL–cancer link.

Mendelian randomization (MR) analysis emerges as a powerful tool for causal inference amidst the proliferation of genome-wide association studies (GWAS) (12), capitalizing on genetic variation as a natural experiment. MR employs genetic markers strongly tied to the exposure as instrumental variables (IVs), mimicking randomized controlled trials by leveraging the random allocation of alleles through Mendelian inheritance, independent of confounders.

Our investigation employed a two-sample MR approach utilizing public GWAS datasets to scrutinize the potential causal associations between UL and a diverse array of cancers. This encompassed low malignant potential and serous ovarian cancers, invasive mucinous and clear cell ovarian cancers, breast, brain, stomach, uterine corpus, thyroid, lung, cervical, bowel, skin melanoma, renal, hematological, and endometrial cancers. Cancer types were chosen based on accessible GWAS data. Clarifying the causal dynamics between UL and these cancers can significantly inform preventive and therapeutic interventions for affected populations.

## Methods

### Study design

**Figure 1:**
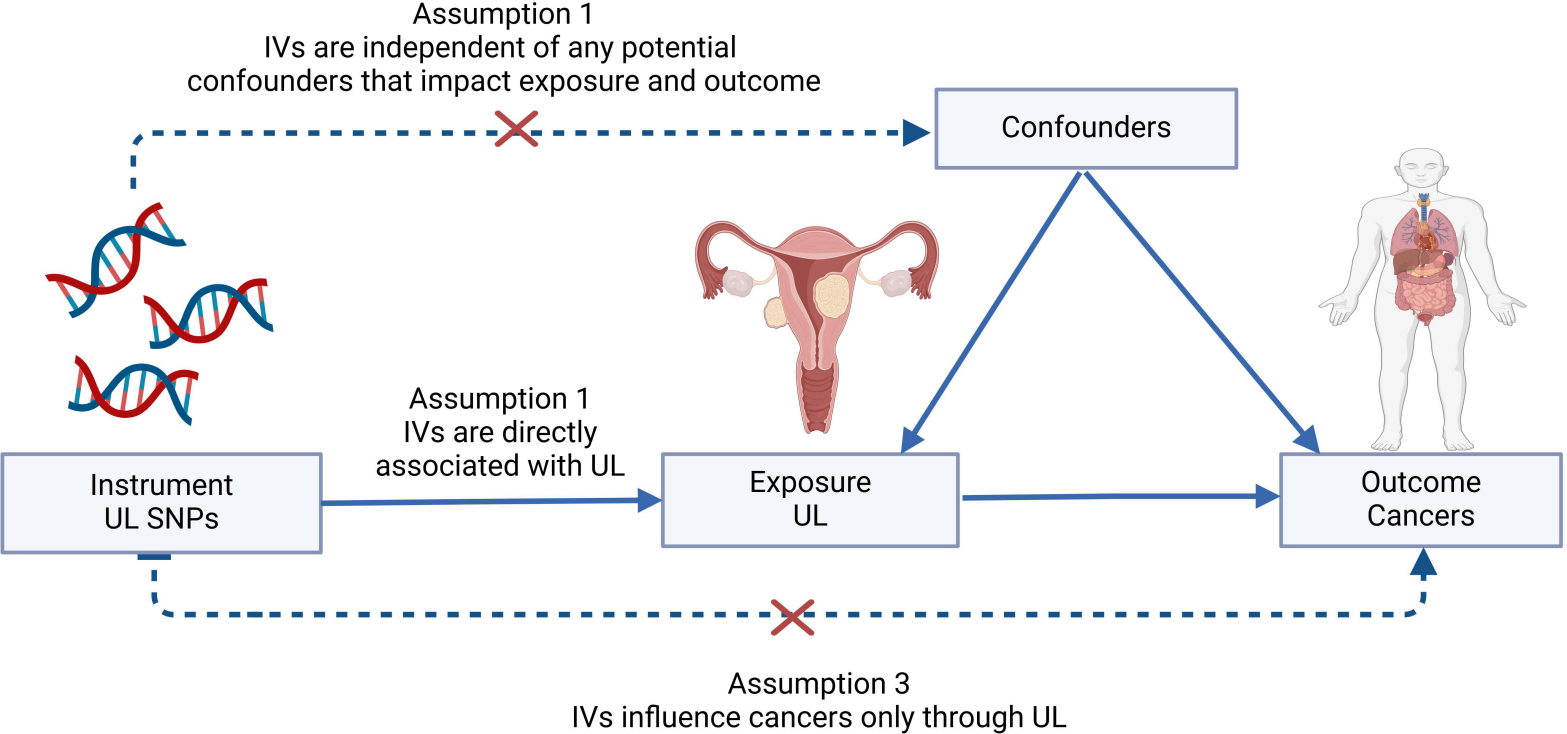
Graphical abstract to show the overview of this study design. The MR framework was based on the three basic Mendelian randomization assumptions. IVs, instrumental variables; SNP, single-nucleotide polymorphism; UL, uterine leiomyoma.

Within the scope of this investigation, we implemented a two-sample MR methodology to estimate the causal associations between UL and 16 frequently occurring site-specific cancers, harnessing genetic IVs in the form of single-nucleotide polymorphisms (SNPs). The validity of the causal inference rested firmly upon adherence to three paramount assumptions: (I) the genetic variants demonstrate a robust correlation with parameters indicative of UL; (II) these genetic markers maintain independence from any confounding elements that might influence the relationship between the exposure (UL) and the outcome (cancers); and (III) the mechanism by which these genetic variants impact cancer susceptibility is solely through their effect on UL-related attributes (13). The essence of a two-sample MR analysis lies in its capacity to discern causal associations between exposures and outcomes derived from distinct or non-overlapping population cohorts. A visual summary outlining the architecture of our study design is depicted in **Figure 1**.

### Data sources

For our analysis, summary statistics for UL were sourced from the FinnGen database (https://r10.finngen.fi/), encompassing 18,060 cases and 105,519 controls of European descent, with UL diagnosis in accordance with ICD-10 code D25. Meanwhile, the comprehensive summary statistics for various cancers under study were retrieved from the Integrative Epidemiology Unit’s (IEU) Open GWAS project repository located at https://gwas.mrcieu.ac.uk. In our MR analysis, the inclusion and exclusion criteria for cases and controls were predetermined in the original GWAS. When utilizing data from publicly available GWAS datasets like those from the FinnGen consortium and the IEU Open GWAS project, the classification of cases and controls is based on the GWAS design criteria regarding diagnosis and individual classification. Typically, participants in these public GWAS datasets are categorized as “cases” (individuals with the specific disease) and “controls” (healthy individuals without the disease manifestation) based on clinical diagnosis, questionnaires, medical records, or biomarker testing. Central to our two-sample MR methodology was the requirement for two discrete sample sets originating from a shared population. To circumvent biases resulting from ethnic stratification, GWAS datasets not representing European ancestry were omitted. Similarly, to minimize potential overlap-induced biases, GWAS datasets for cancer that incorporated subjects also present in the FinnGen database were excluded. Following these rigorous selection criteria, we procured GWAS data for 16 prevalent site-specific cancers from the IEU Open GWAS resource. In our study, ovarian cancer was categorized into subtypes including low malignant potential ovarian cancer, serous ovarian cancer, invasive mucinous ovarian cancer, and clear cell ovarian cancer. Conversely, cancers such as breast, lung, and brain malignancies were examined as overarching categories without subtype differentiation. An exhaustive summary of our dataset origins and specifics is provided in **Supplementary Table 1**, affirming the exclusive use of European ancestry participants throughout our investigation. Given the public accessibility of the datasets employed, no additional ethical permissions or individual consent processes beyond those already obtained by the respective GWAS initiatives were deemed necessary.

### IVs selection

To ensure the rigor of our analysis, we applied SNPs that surpassed the genome-wide significance level (*p*-value < 5 × 10^−8^) to serve as IVs for UL. To uphold data integrity, we enforced strict parameters to avoid linkage disequilibrium (LD): a window size of 10 kilobases (kb = 10,000) and an LD threshold of *r* ^2^ = 0.001. Additionally, we required an odds ratio (OR) between SNPs and the exposure to exceed 5 × 10^−5^. The strength of each IV was assessed using *F*-statistics, with values greater than 10 indicating a robust correlation between IVs and UL. This *F*-statistic is mathematically represented as *F* = (*R* ^2^ × (*N* − 2))/((1 − *R* ^2^) × (1 − *R* ^2^)) (14), confirming the robustness of the genetic instruments. To ascertain the directional causality from UL to the cancer outcomes, we employed the Steiger filtering test. Initially, 75 candidate SNPs were shortlisted, but after applying these rigorous filters and standardizing alleles across datasets, a final set of 12 to 70 independent, common SNPs were retained as valid IVs for the cancers studied. The complete list of SNPs implicated in UL is detailed in **Supplementary Table 2**.

### Statistical analysis

Statistically, our primary analytical tool was the fixed-effect inverse variance weighted (IVW) method to discern potential causal effects connecting UL and the 16 cancer outcomes (15). In instances where the Cochrane *Q* statistic revealed significant heterogeneity (*p* < 0.05), we employed the multiplicative random-effects IVW model to adjust for such variability (16). Additionally, we adopted three **Supplementary Methods** including MR-Egger, weighted median, and weighted mode to evaluate the robustness of the IVW results comprehensively. The IVW method has the strongest power to detect relationships between exposures and outcomes (17). The MR-Egger method uniquely enables the derivation of a corrected causal effect estimate, maintaining unbiasedness even under the scenario where not all chosen IVs adhere to the strictest validity criteria (18). Conversely, the weighted median approach furnishes more resilient effect estimates by adopting a less stringent assumption that merely requires at least half of the employed IVs to be genuinely instrumental (19). The weighted mode method, on the other hand, rests its dependability on the premise that the most prominent group of IVs sharing comparable causal impacts constitutes a valid set.

Furthermore, we implemented sensitivity analyses to validate the causal effect estimates, including the random-effect IVW for heterogeneity correction (16), Cochrane *Q* for assessing instrument variability (19), and MR-Egger intercept and MR-PRESSO global test for pleiotropy detection. A combination of MR-Egger and MR-PRESSO allowed for pleiotropy identification and correction, with a *p*-value above 0.05 indicating an absence of horizontal pleiotropy (20). The leave-one-out analysis was also carried out to identify influential outliers. We augmented our methodology by integrating advanced techniques such as the contamination mixture method (ConMix), robust adjusted profile score (RAPS), debiased inverse-variance weighted method (DIVW), and constrained maximum likelihood (CML) to fortify our results. All statistical procedures were executed using the TwoSampleMR and MR-PRESSO packages within the R software environment (version 4.3.0), adhering to the guidelines outlined in the STROBE-MR statement for transparent reporting of MR studies in epidemiology (21).

## Results

In adherence to the outlined criteria, we meticulously selected SNPs from our exposure datasets. This process yielded a total of 12 to 70 SNPs related to UL exposure across 16 types of cancer. Notably, all IVs demonstrated *F*-statistics exceeding 10, a robust indicator of no detectable bias, and a fulfillment of the first assumption inherent to MR studies. Leveraging these SNPs as IVs, we embarked on a comprehensive MR analysis to probe into the causal associations between UL exposure and the aforementioned 16 cancers.

**Figure 2:**
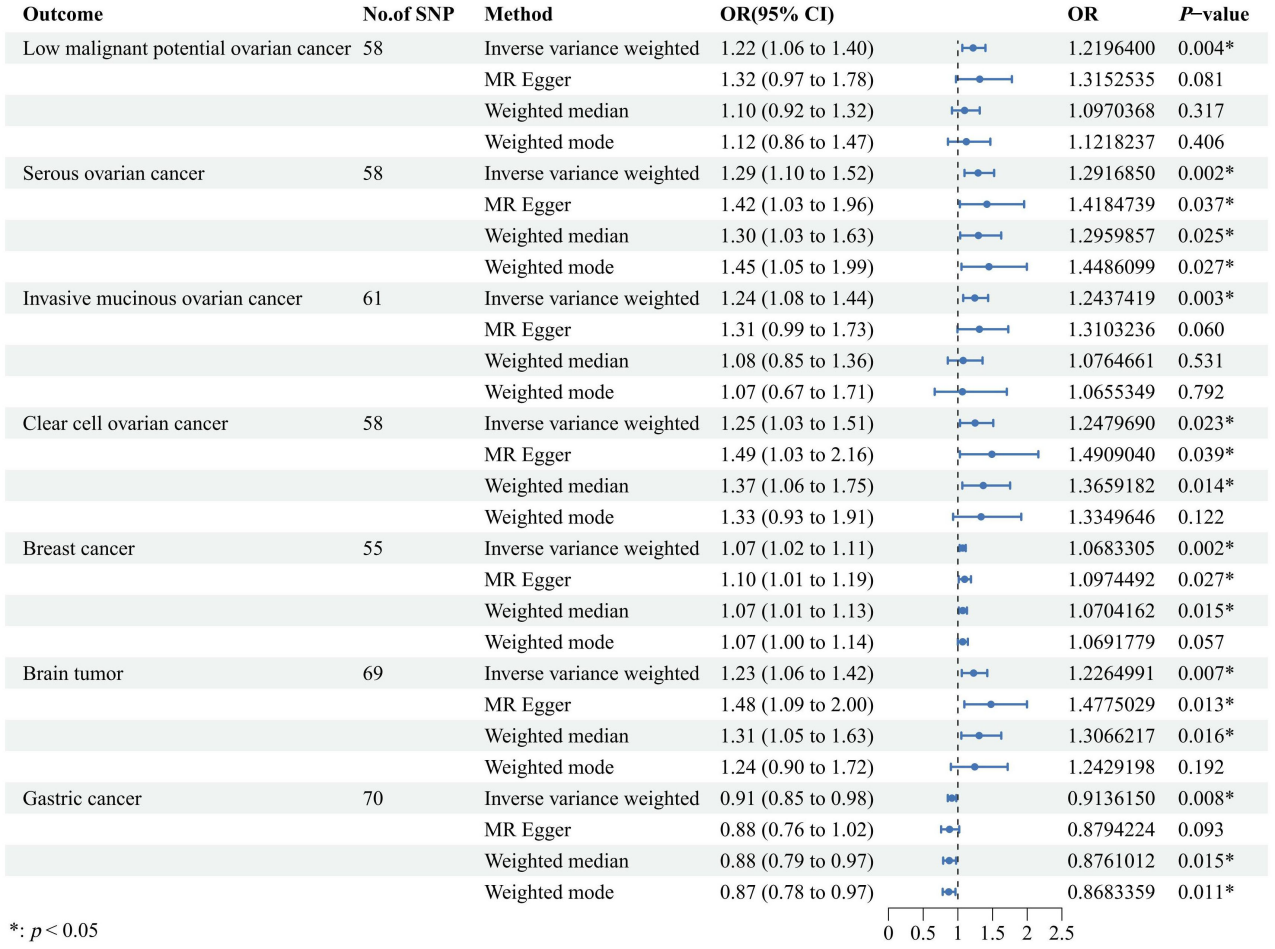
A forest plot designed to visually illustrate the causal effects of uterine leiomyoma on a selection of cancers, encompassing low malignant potential ovarian cancer, serous ovarian cancer, invasive mucinous ovarian cancer, clear cell ovarian cancer, breast cancer, brain tumor, and gastric cancer. The primary analytical strategy employed was the random-effects inverse variance-weighted method, complemented by three additional approaches, including MR-Egger, weighted median, and weighted mode, to corroborate the robustness of our findings. OR, odds ratio; CI, confidence interval; SNP, single-nucleotide polymorphism; MR, Mendelian randomization.

**Figure 3:**
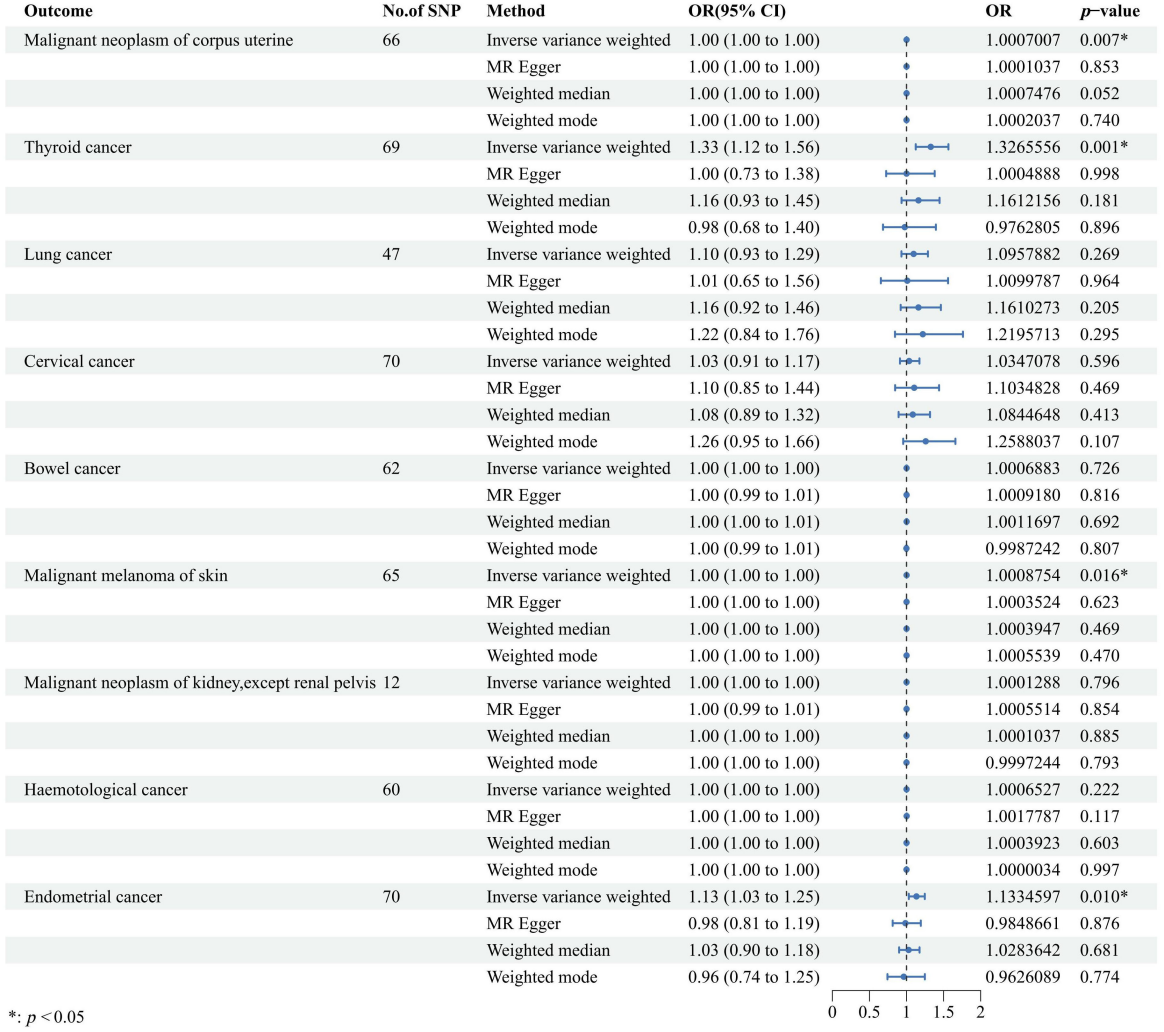
Forest plot to visualize the causal effects of uterine leiomyoma on cancers including malignant neoplasm of corpus uterine, thyroid cancer, lung cancer, cervical cancer, bowel cancer, malignant melanoma of skin, malignant neoplasm of kidney, hematological cancer, and endometrial cancer. The random-effects inverse variance weighted method served as the primary approach, while three other methods, namely, MR-Egger, weighted median, and weighted mode, were applied as auxiliary methods. OR, odds ratio; CI, confidence interval; SNP, single-nucleotide polymorphism; MR, Mendelian randomization.

Our findings revealed a significant association where each standard deviation (SD) increase in genetically predicted UL exposure corresponded to an increased OR for various cancers. Specifically, ovarian cancer showed a 22% increase in risk [OR = 1.22, 95% confidence interval (CI): 1.06–1.40, *p* = 0.004], serous ovarian cancer showed a 29% increase in risk (OR = 1.29, 95% CI: 1.10–1.52, *p* = 0.002), invasive mucinous ovarian cancer showed a 24% increase in risk (OR = 1.24, 95% CI: 1.08–1.44, *p* = 0.003), clear cell ovarian cancer showed a 25% increase in risk (OR = 1.25, 95% CI: 1.03–1.51, *p* = 0.023), breast cancer showed a 7% increase in risk (OR = 1.07, 95% CI: 1.02–1.11, *p* = 0.002), and brain tumors showed a 23% increase in risk (OR = 1.23, 95% CI: 1.06–1.42, *p* = 0.007). Conversely, gastric cancer showed a 9% decrease in risk (OR = 0.91, 95% CI: 0.85–0.98, *p* = 0.008). Moreover, similar results were also observed in the three other methods to estimate the relationships between UL and cancer risks. These findings are graphically represented in **Figure 2** and detailed numerically in **Supplementary Table 3**. However, our analysis did not uncover any causal associations between genetically predicted UL exposure and the risk of malignant neoplasms of the corpus uteri, thyroid, lung, cervix, bowel, skin melanoma, kidney (excluding renal pelvis), or endometrial cancer (**Figure 3**). To enhance visual comprehension, we plotted scatter graphs illustrating the causal impact of all IVs on both exposure and outcome variables. A positive gradient within these plots denoted a negative correlation between exposure and outcome, while a negative gradient indicated a positive correlation. **Supplementary Figure 1** provides these scatter plots for each cancer type.

In our sensitivity analyses, Cochrane *Q* test revealed heterogeneity (*p* < 0.05) in the relationship between UL and low malignant potential ovarian cancer, serous ovarian cancer, clear cell ovarian cancer, and breast cancer. To address this, we employed the multiplicative random-effect IVW method, which effectively corrected for heterogeneity. No evidence of directional pleiotropy was detected upon reanalysis using the MR-Egger intercept approach (**Supplementary Figure 2**, **Supplementary Table 4**), nor were any outlier SNPs identified via MR-PRESSO. Moreover, leave-one-out analyses suggested that the causal estimates remained robust against the influence of individual SNPs (**Supplementary Figures 3–18**). Importantly, the results obtained from the four additional methods, including ConMix, RAPS, DIVW, and CML, are consistent with the IVW results (**Supplementary Figures 19, 20**).

## Discussion

Our study represents a pioneering effort in utilizing the two-sample MR method to explore the causal associations between genetic predisposition to UL exposure and 16 site-specific cancers. We provide evidence suggesting that individuals with a higher genetic susceptibility to UL are at an elevated risk for developing low malignant potential ovarian cancer, serous ovarian cancer, invasive mucinous ovarian cancer, clear cell ovarian cancer, breast cancer, and brain tumors. Intriguingly, this same genetic vulnerability appears to correlate with a decreased likelihood of gastric cancer. However, our findings do not support a causal link between genetic predisposition to UL and the incidence of malignant neoplasms in the corpus uteri, thyroid, lung, cervix, bowel, skin melanoma, kidney (excluding the renal pelvis), hematological malignancies, or endometrial cancer. These results underscore the complex interplay between genetic factors, environmental exposures, and cancer development, highlighting the need for further research to fully elucidate these relationships.

Given its prevalence as the most frequently encountered benign tumor in female patients, UL has garnered attention in recent investigations concerning its potential implications for subsequent cancer risk. Several observational studies are consistent with our finding. For instance, a population-based case–control study, encompassing 4,088 ovarian cancer cases and 16,348 controls, documented a heightened risk of ovarian cancer among individuals with a history of UL exposure relative to healthy counterparts (7). Similarly, another comprehensive nationwide study revealed a slight elevation in breast cancer risk (OR = 1.14, 95% CI: 1.07–1.21) among women with UL, with the association persisting even after accounting for hormonal factors (8). Additionally, a retrospective cohort study on a national scale highlighted that women exposed to UL exhibited a greater propensity to develop meningioma, with a particularly pronounced effect observed in the age bracket of 35 to 65 years (10). Our MR study not only aligns with these observational findings but also fortifies the causal inference linking UL to increased risks of ovarian cancer, breast cancer, and meningioma. Yet, it is noteworthy that some studies have yielded contrasting results. Lauren et al. posited that UL exposure history bears no significant correlation with the overall incidence of breast cancer (11), underscoring the complexity and variability of outcomes across different studies. It is worth noting that we observed an inverse causal association between UL and gastric cancer. So far, there is little evidence on the association between UL and gastric cancer. Consequently, there exists a clear imperative for additional studies to further delineate and understand the nature of this relationship, potentially shedding new light on the mechanisms underlying the development of gastric cancer in the context of UL exposure.

Some underlying mechanisms may be proposed to support the positive effect of UL on the heightened risk of ovarian cancer, breast cancer, and brain tumor. First, sex hormones, notably estrogen and progesterone, are integral to the pathogenesis of both UL and certain hormone-sensitive malignancies. It is now widely recognized that these hormones play a pivotal role in the occurrence and progression of UL. Englund and colleagues demonstrated that UL tissues exhibit a marked upregulation of estrogen and progesterone receptors when compared to corresponding myometrial tissues (22). Factors that extend the duration of estrogen exposure throughout life, such as obesity, early onset of menarche, and delayed menopause, have been shown to increase the prevalence of UL. Oral gonadotropin-releasing hormone (GnRH) antagonists can treat UL by inhibiting the secretion of estrogen (23). A series of studies found that women with high estrogen levels had a significantly higher prevalence of ovarian cancer (24), breast cancer (25, 26), and brain tumor (27), reinforcing the notion that hormonal stimulation may contribute to the development of these malignancies. This body of evidence supports the potential mechanism by which hormone-driven processes might influence the etiology of certain cancer types, particularly those responsive to estrogen and progesterone. Second, the dysfunction of the immune system, leading to deregulated cellular proliferation, presents another plausible explanation for the observed phenomena. Recent researchers have highlighted the profound immune system disruption in UL patients (28). For instance, in ovarian cancer, mesothelial cells facilitate metastasis and chemotherapy resistance through their interactions with cancer cells, whereas tumor-associated macrophages play a role in exhibiting protumor or antitumor (29). In breast cancer, the immune system’s influence on disease progression and therapeutic resistance is well-established (30). Glioma-associated macrophages and microglia are crucial in regulating tumor growth, invasion, and recurrence (31). The unregulated immune system in UL may be one of the causes to the prevalence of cancers. In addition, inflammatory response has been thought to be changed during the formation of UL (32), and inflammation also has a complex connection with cancers (33).

Overall, our study revealed positive causal associations between UL and low malignant potential ovarian cancer, serous ovarian cancer, invasive mucinous ovarian cancer, clear cell ovarian cancer, breast cancer, and brain tumor. While the precise underlying mechanisms remain to be fully elucidated, it may provide a new perspective to explore the mechanism of cancers, especially in women with UL. It also needs further study to verify whether surgical removal of UL can prevent the development of certain cancers. Our results remind healthcare professionals that screening for ovarian cancer, breast cancer, and brain tumors regularly in women with a history of UL may contribute to early diagnosis and treatment of these diseases.

We identified several strengths in our study. Firstly, we leveraged randomly allocated genetic variants as IVs to mitigate reverse causality and potential confounding bias, which is a superior approach compared to observational studies. Secondly, we comprehensively examined a wide range of cancers previously reported to be associated with UL for MR analysis, thereby facilitating a thorough exploration of the causal relationships between UL and site-specific cancers. This comprehensive analysis offers a more holistic understanding of the potential carcinogenic effects of UL. Thirdly, we reinforced the validity of our findings through the application of some distinct sensitivity analysis methods, ensuring the robustness of our results. Although we did encounter heterogeneity among the IVs in certain analyses, the absence of evidence for horizontal pleiotropy as indicated by the MR-Egger intercept lends credibility to our findings, minimizing the risk of biased outcomes. However, we must acknowledge several limitations of this study. Firstly, as two-sample MR necessitates both samples to originate from the same population, our study exclusively featured participants of European descent, thus requiring further research to generalize these results to other populations. Secondly, the sample size of some cancer-related datasets in this study was small, potentially rendering them unrepresentative. Thirdly, while MR analysis is a powerful tool for inferring causal effects, it falls short in elucidating the intricate biological mechanisms underpinning the association between UL exposure and cancer development. Further investigation, including experimental and clinical studies, is indispensable to unravel the complex interplay between UL and cancer etiology, thereby substantiating our hypothesis that UL may exert a positive influence on cancer progression.

## Conclusion

In conclusion, our study contributes valuable insights into the potential causal links between UL and cancer, though it is essential to acknowledge the need for additional research to validate and expand upon these preliminary findings. Future endeavors should aim to overcome the limitations identified here, paving the way for a deeper understanding of the role of UL in cancer development and the identification of effective preventive measures.

## Data Availability

The original contributions presented in the study are included in the article/**Supplementary Material**. Further inquiries can be directed to the corresponding authors.
