# Supplementary material for: Causal relationship between genetically predicted uterine leiomyoma and cancer risk: a two-sample Mendelian randomization
Source: Front Endocrinol (Lausanne). 2024 Aug 29;15:1429165. doi: 10.3389/fendo.2024.1429165 (PMC11390398; doi:10.3389/fendo.2024.1429165)
Supplement: Supplementary file 1 [file DataSheet1.pdf]

Supplementary Figures

Figure S1: Scatter plot to visualize the causal effect of uterine leiomyoma on cancers................. 2
Figure S2: Funnel plot to visualize the causal effect of uterine leiomyoma on cancers................. 3
Figure S3: Leave-one-out inverse-variance weighted mendelian randomization analyses of uterine leiomyoma on low malignant potential ovarian cancer.. 4
Figure S4: Leave-one-out inverse-variance weighted mendelian randomization analyses of uterine leiomyoma on serous ovarian cancer..... 5
Figure S5: Leave-one-out inverse-variance weighted mendelian randomization analyses of uterine leiomyoma on invasive mucinous ovarian cancer 6
Figure S6: Leave-one-out inverse-variance weighted mendelian randomization analyses of uterine leiomyoma on clear cell ovarian cancer... 7
Figure S7: Leave-one-out inverse-variance weighted mendelian randomization analyses of uterine leiomyoma on breast cancer 8
Figure S8: Leave-one-out inverse-variance weighted mendelian randomization analyses of uterine leiomyoma on brain tumor... 9
Figure S9: Leave-one-out inverse-variance weighted mendelian randomization analyses of uterine leiomyoma on gastric cancer... 10
Figure S10: Leave-one-out inverse-variance weighted mendelian randomization analyses of uterine leiomyoma on malignant neoplasm of corpus uterine... 11
Figure S11: Leave-one-out inverse-variance weighted mendelian randomization analyses of uterine leiomyoma on thyroid cancer... 12
Figure S12: Leave-one-out inverse-variance weighted mendelian randomization analyses of uterine leiomyoma on lung cancer 13
Figure S13: Leave-one-out inverse-variance weighted mendelian randomization analyses of uterine leiomyoma on cervical cancer.. 14
Figure S14: Leave-one-out inverse-variance weighted mendelian randomization analyses of uterine leiomyoma on bowel cancer... 15
1Figure S15: Leave-one-out inverse-variance weighted mendelian randomization analyses of uterine leiomyoma on malignant melanoma of skin... 16
Figure S16: Leave-one-out inverse-variance weighted mendelian randomization analyses of uterine leiomyoma on malignant neoplasm of kidney... 17
Figure S17: Leave-one-out inverse-variance weighted mendelian randomization analyses of uterine leiomyoma on haemotological cancer.. 18
Figure S18: Leave-one-out inverse-variance weighted mendelian randomization analyses of uterine leiomyoma on endometrial cancer...19
Figure S19: Forest plot of ConMix, RAPS, DIVW and CML methods to visualize the casual effect of uterine leiomyoma on seven cancers with positive results..20
Figure S20: Forest plot of ConMix, RAPS, DIVW and CML methods to visualize the casual effect of uterine leiomyoma on six other cancers with negative results... 21

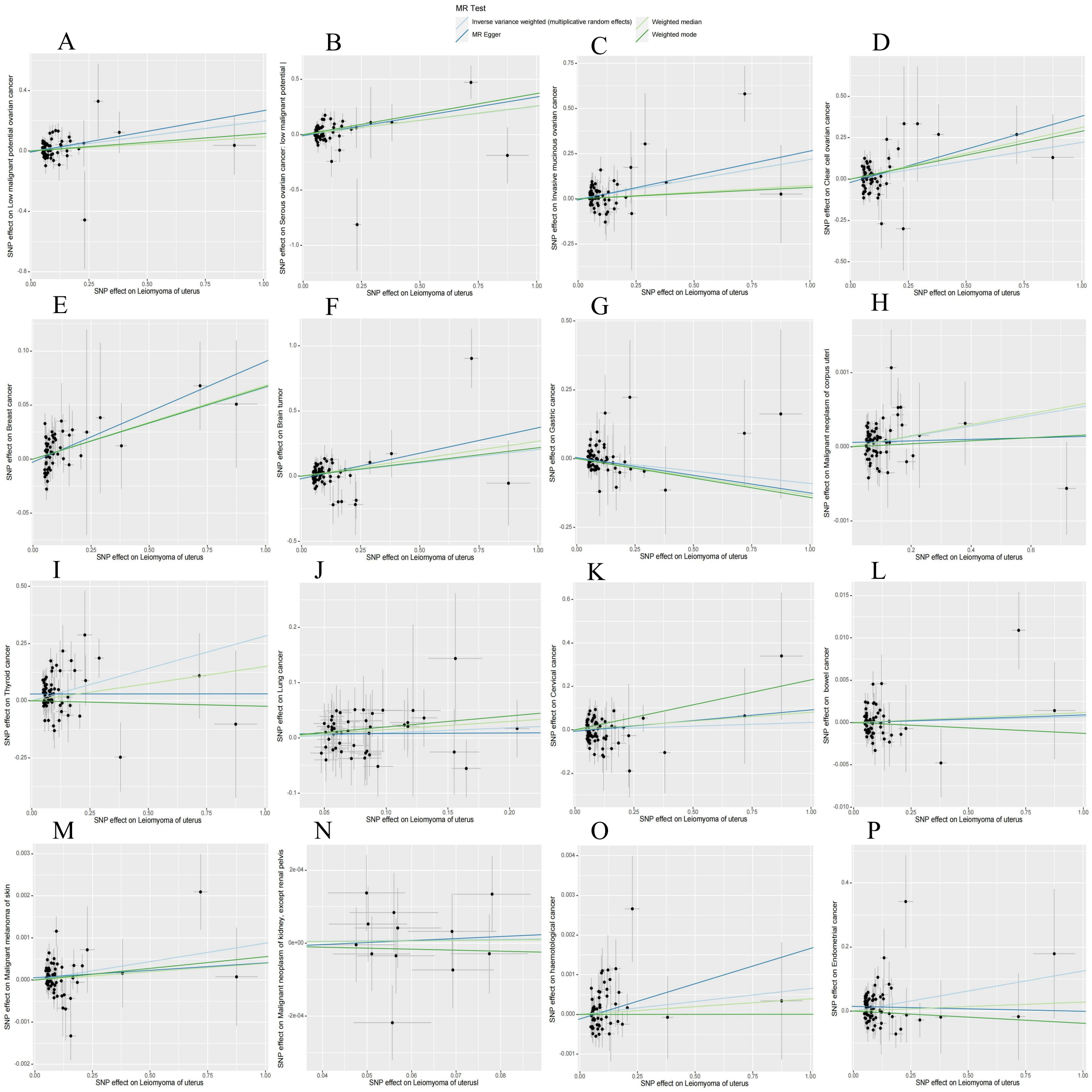

Figure S1. Scatter plot to visualize the causal effect of uterine leiomyoma on cancers. A, Low malignant potential ovarian cancer; B, Serous ovarian cancer; C, Invasive mucinous ovarian cancer; D, Clear cell ovarian cancer; E, Breast cancer; F, Brain tumor; G, Gastric cancer; H, Malignant neoplasm of corpus uterine; I, Thyroid cancer; J, Lung cancer; K, Cervical cancer; L, Bowel cancer; M, Malignant melanoma of skin; N, Malignant neoplasm of kidney; O, Haemotological cancer; P, Endometrial cancer.

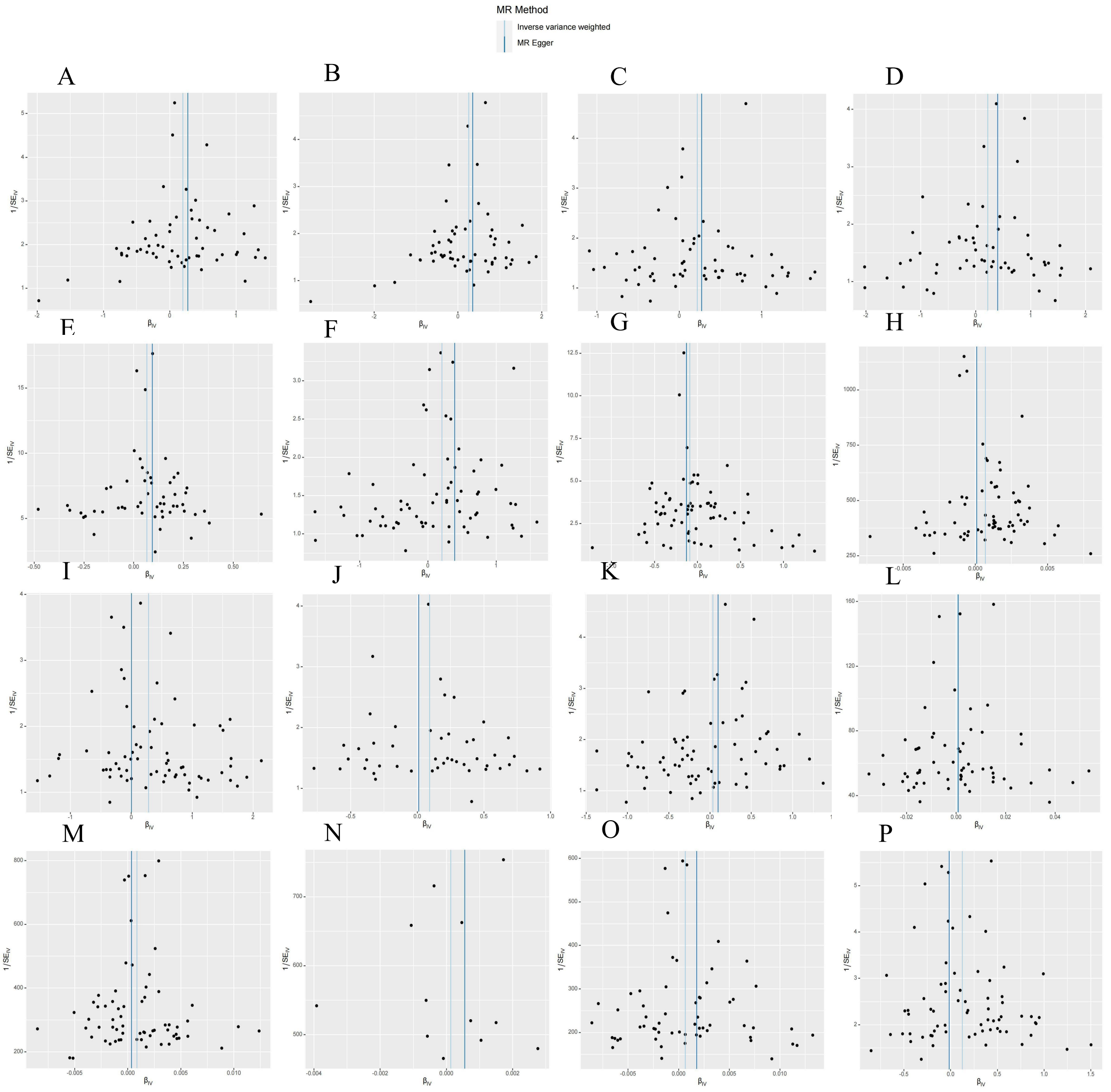

Figure S2. Funnel plot to visualize the causal effect of uterine leiomyoma on cancers. A, Low malignant potential ovarian cancer; B, Serous ovarian cancer; C, Invasive mucinous ovarian cancer; D, Clear cell ovarian cancer; E, Breast cancer; F, Brain tumor; G, Gastric cancer; H, Malignant neoplasm of corpus uterine; I, Thyroid cancer; J, Lung cancer; K, Cervical cancer; L, Bowel cancer; M, Malignant melanoma of skin; N, Malignant neoplasm of kidney; O, Haemotological cancer; P, Endometrial cancer.

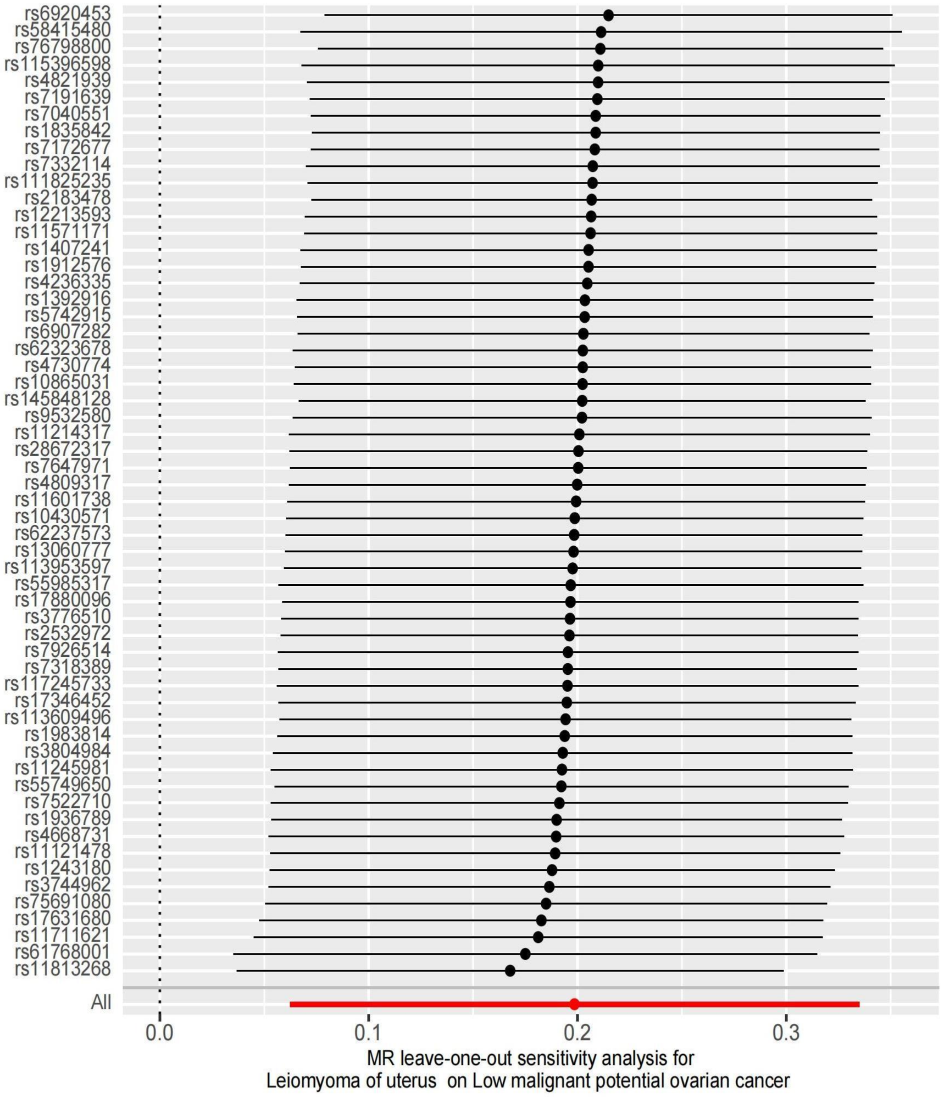

Figure S3. Leave-one-out inverse-variance weighted mendelian randomization analyses of uterine leiomyoma on low malignant potential ovarian cancer.

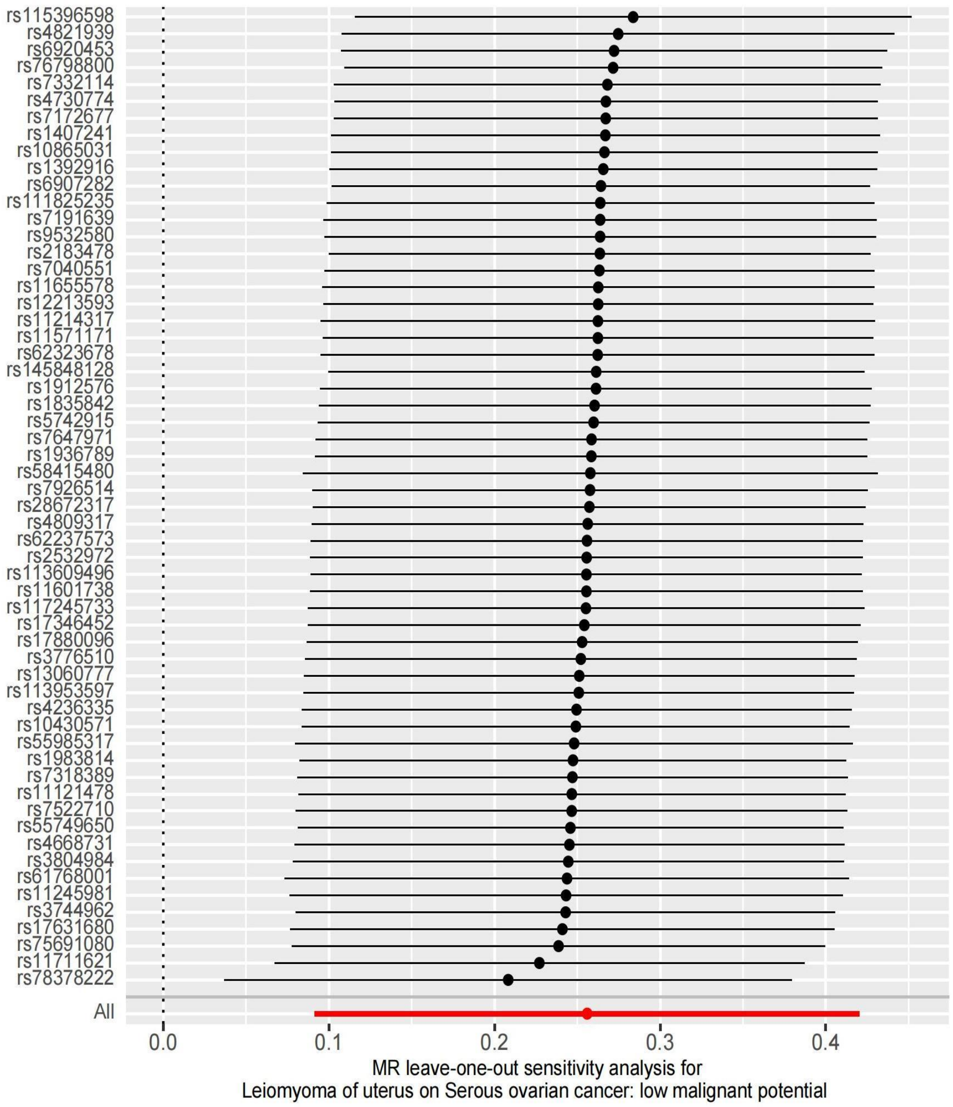

Figure S4. Leave-one-out inverse-variance weighted mendelian randomization analyses of uterine leiomyoma on serous ovarian cancer.

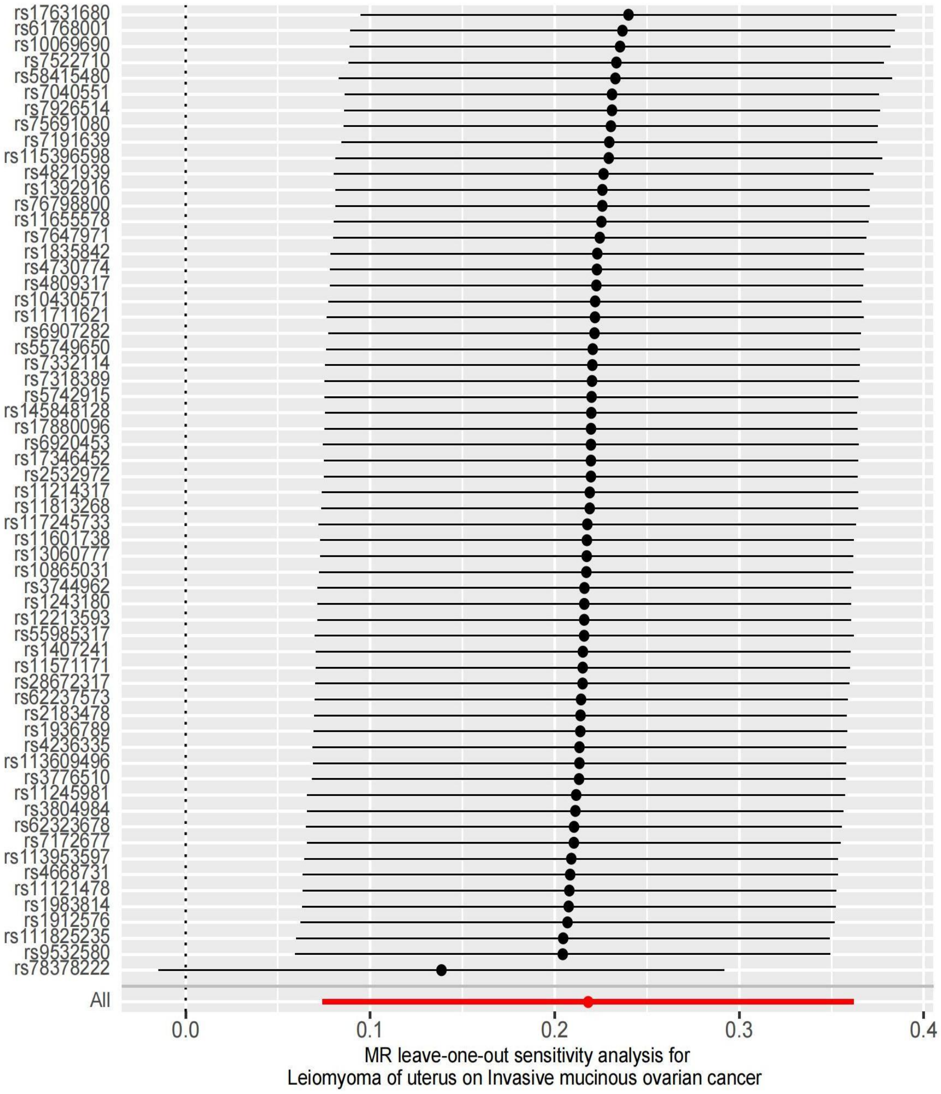

Figure S5. Leave-one-out inverse-variance weighted mendelian randomization analyses of uterine leiomyoma on invasive mucinous ovarian cancer.

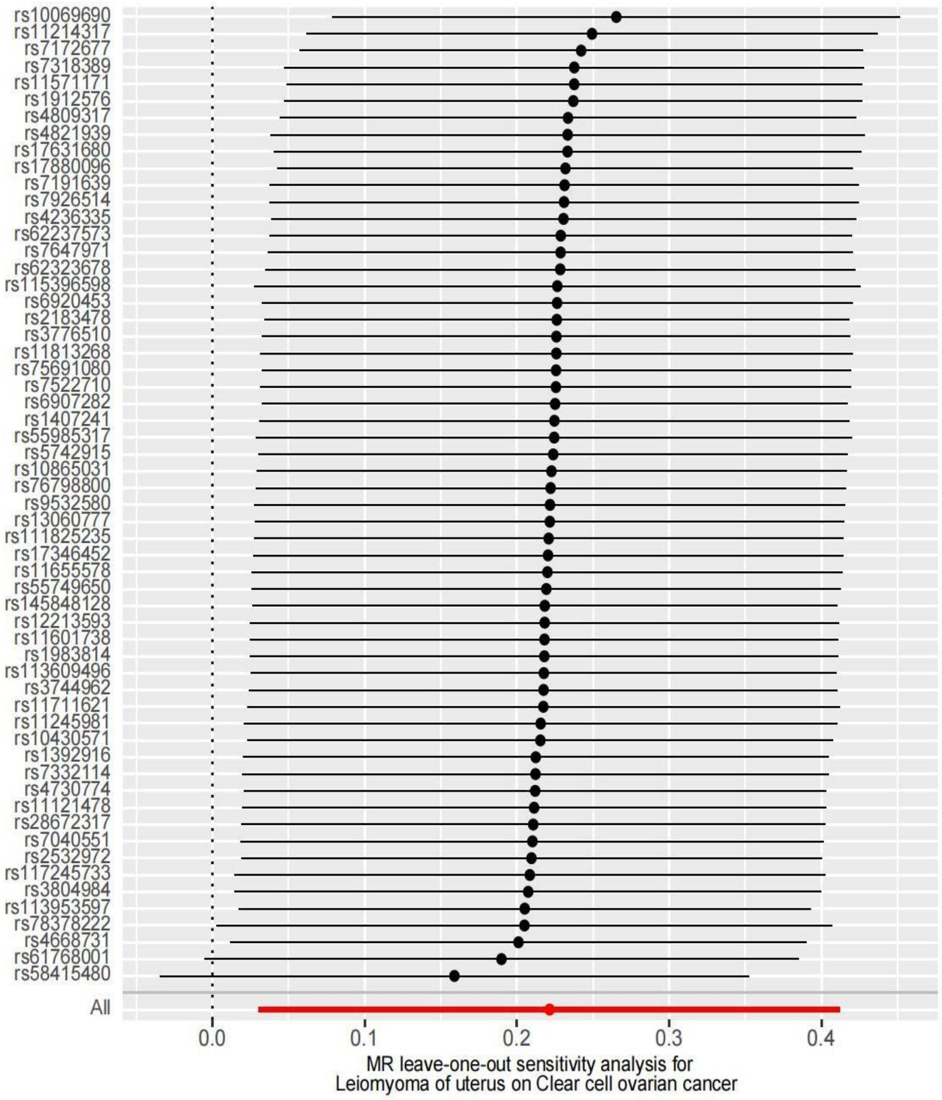

Figure S6. Leave-one-out inverse-variance weighted mendelian randomization analyses of uterine leiomyoma on clear cell ovarian cancer

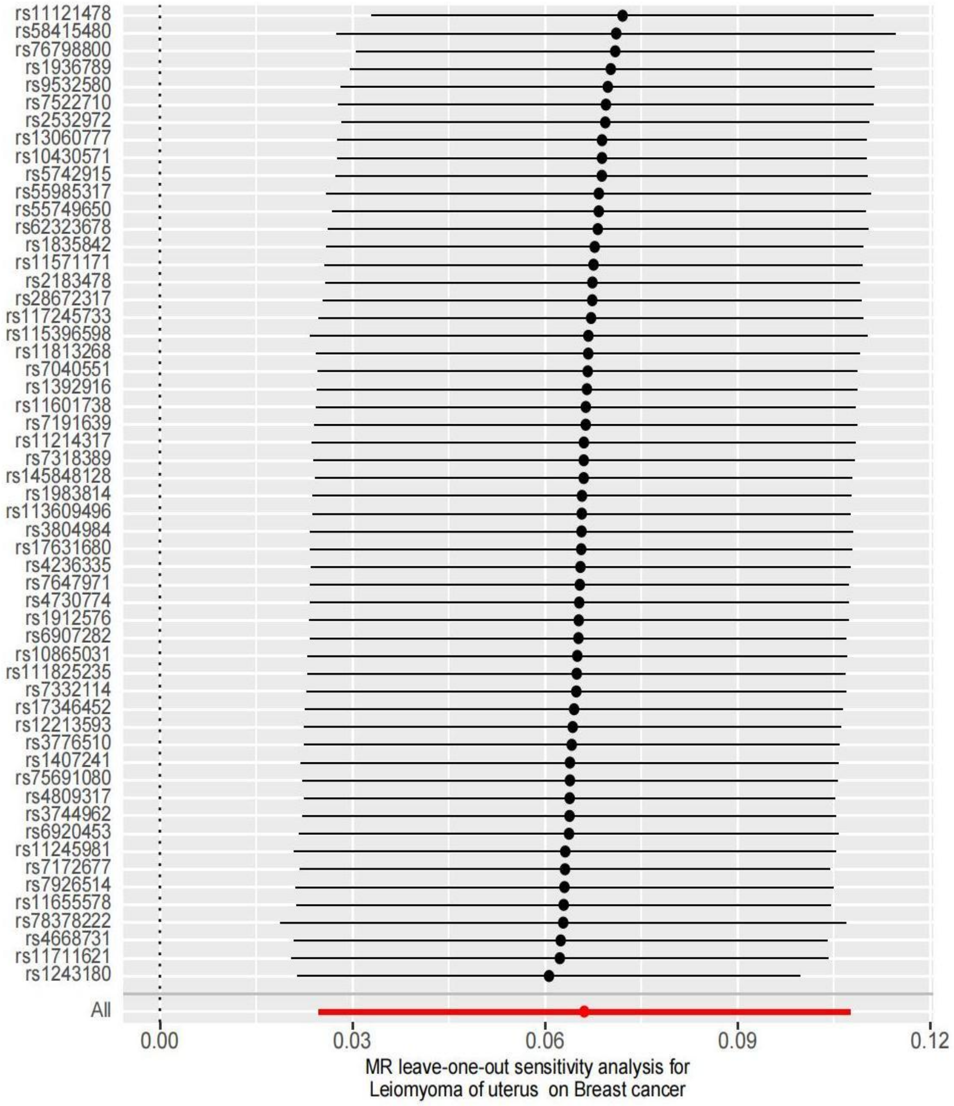

Figure S7. Leave-one-out inverse-variance weighted mendelian randomization analyses of uterine leiomyoma on breast cancer

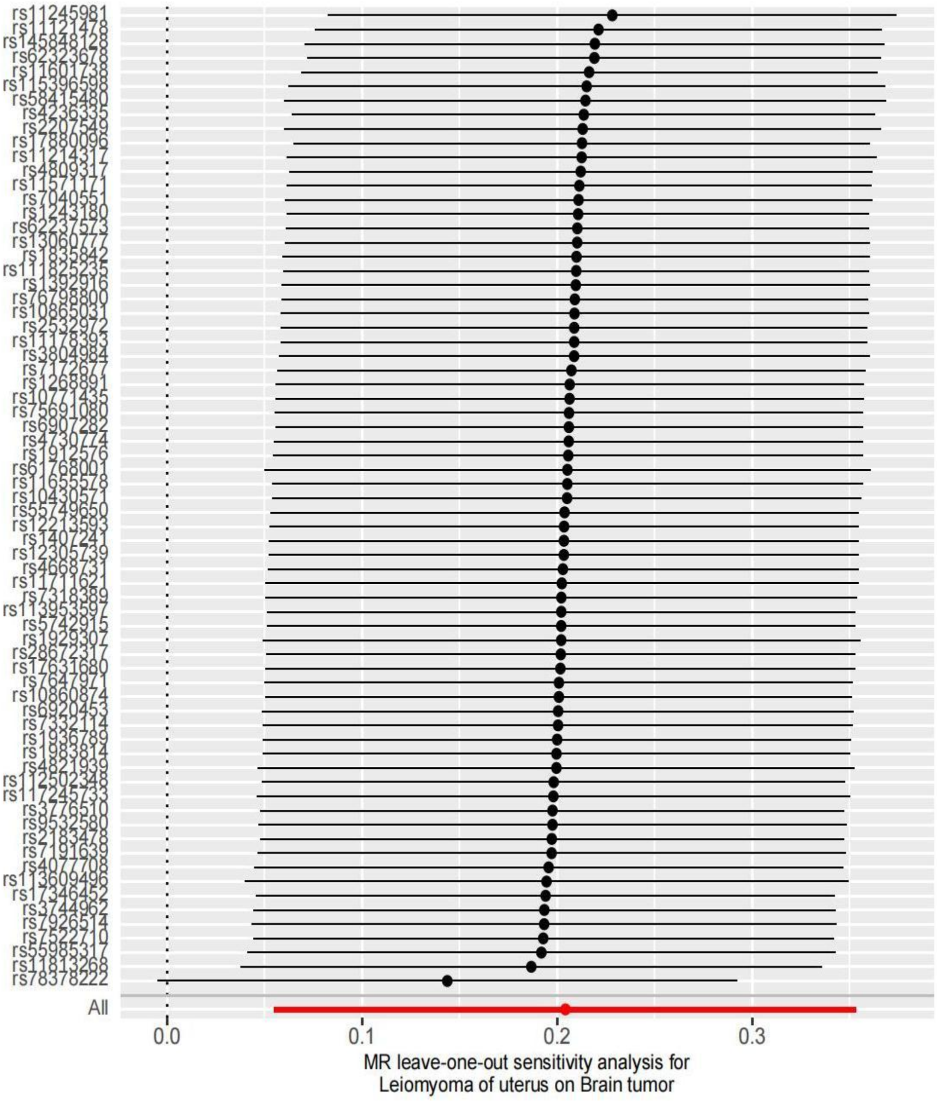

Figure S8. Leave-one-out inverse-variance weighted mendelian randomization analyses of uterine leiomyoma on brain tumor.

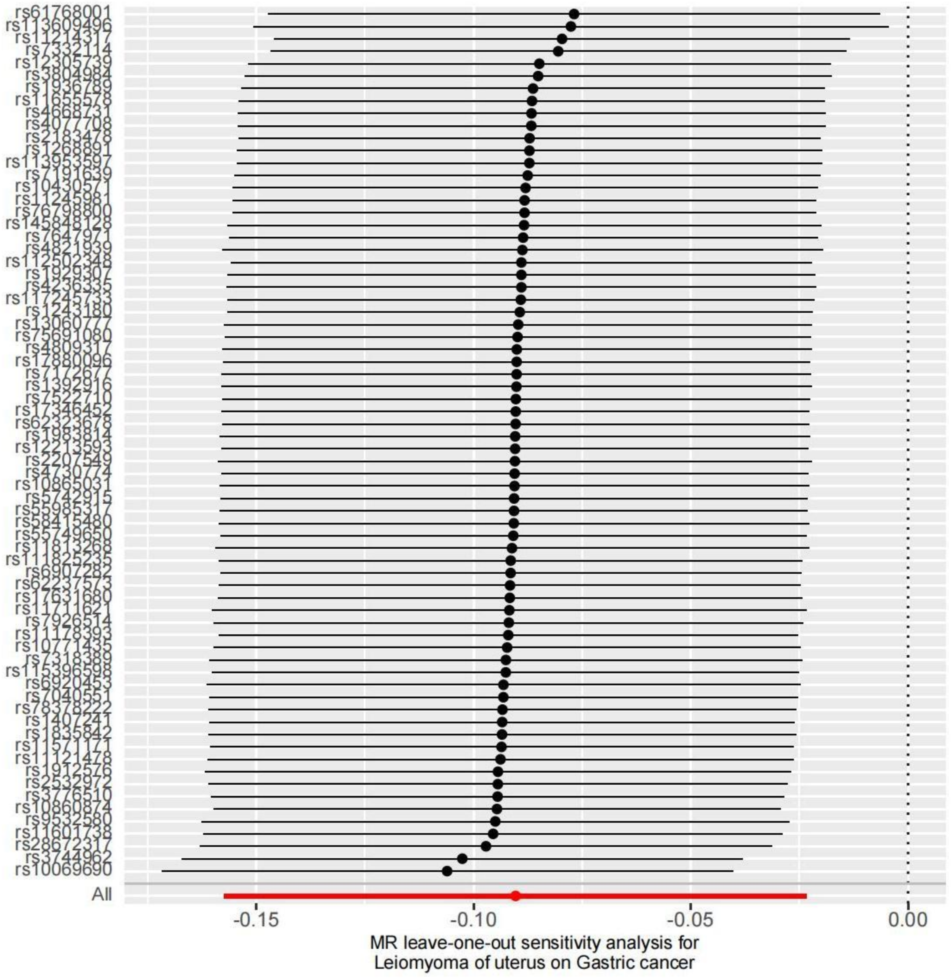

Figure S9. Leave-one-out inverse-variance weighted mendelian randomization analyses of uterine leiomyoma on gastric cancer.

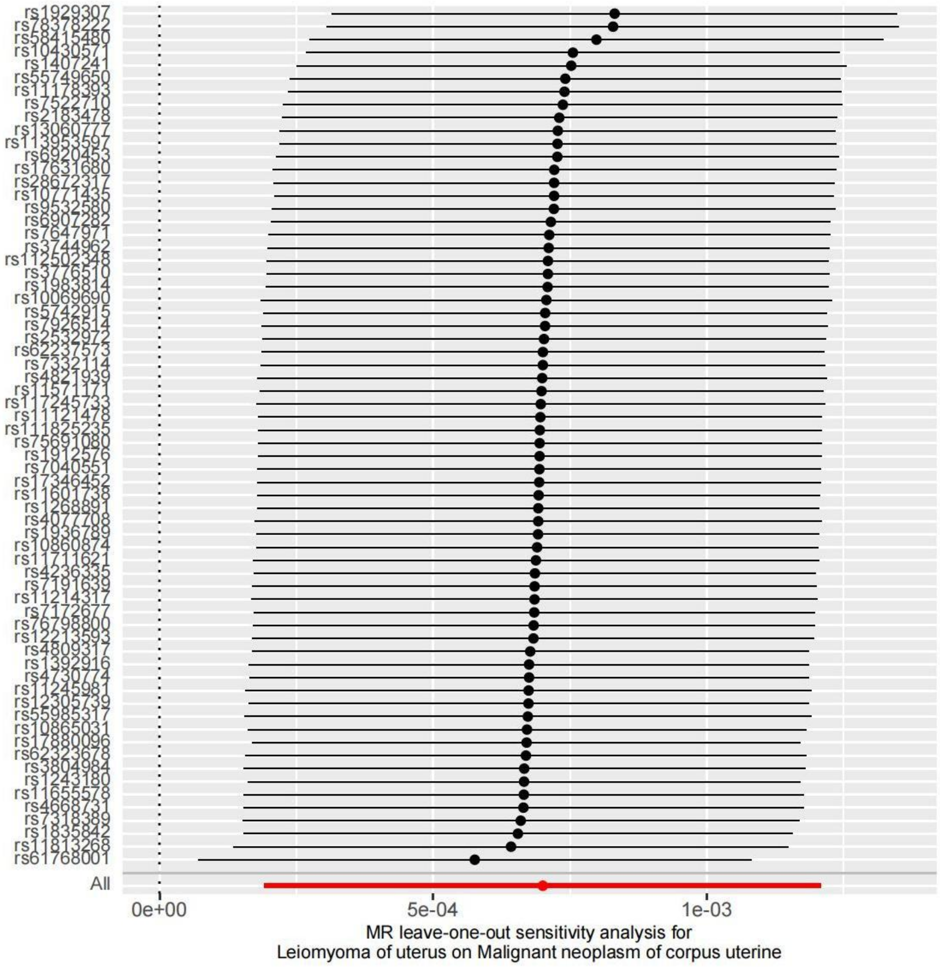

Figure S10. Leave-one-out inverse-variance weighted mendelian randomization analyses of uterine leiomyoma on malignant neoplasm of corpus uterine.

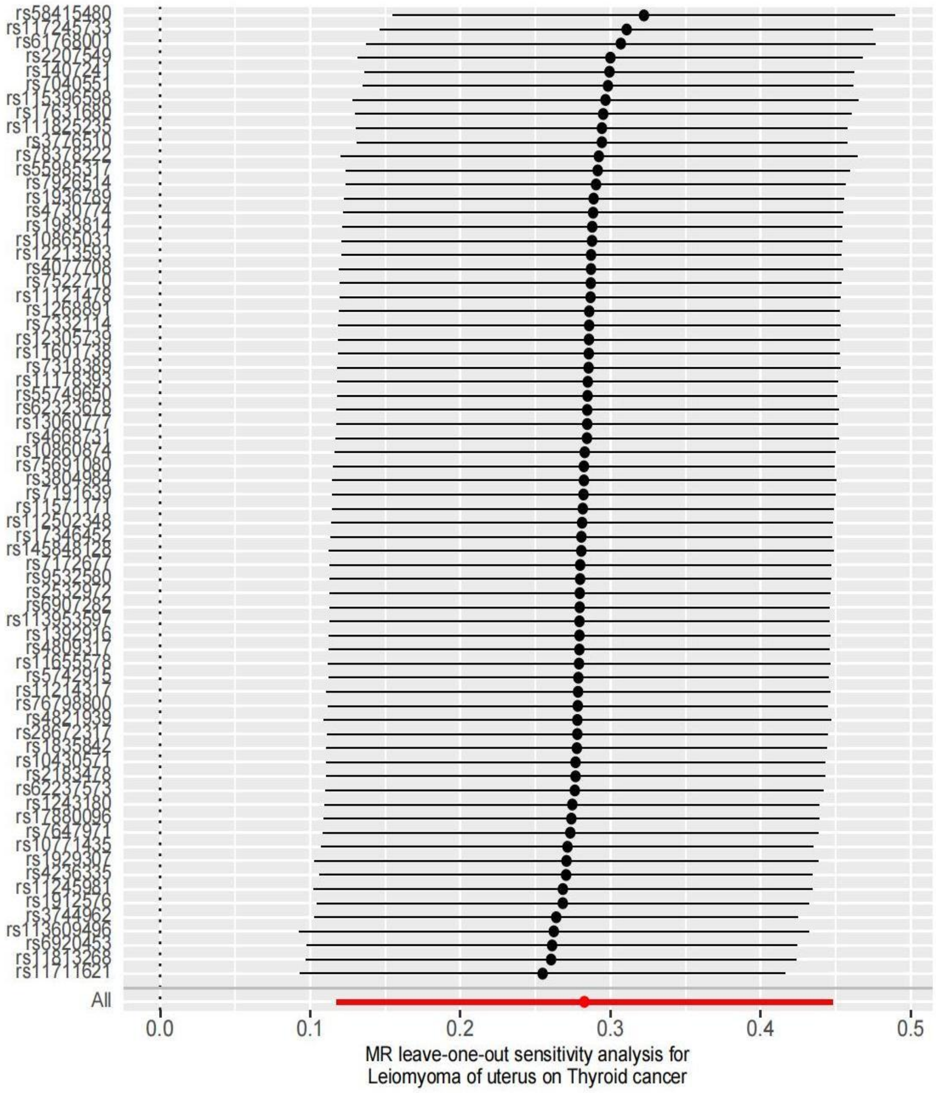

Figure S11. Leave-one-out inverse-variance weighted mendelian randomization analyses of uterine leiomyoma on thyroid cancer.

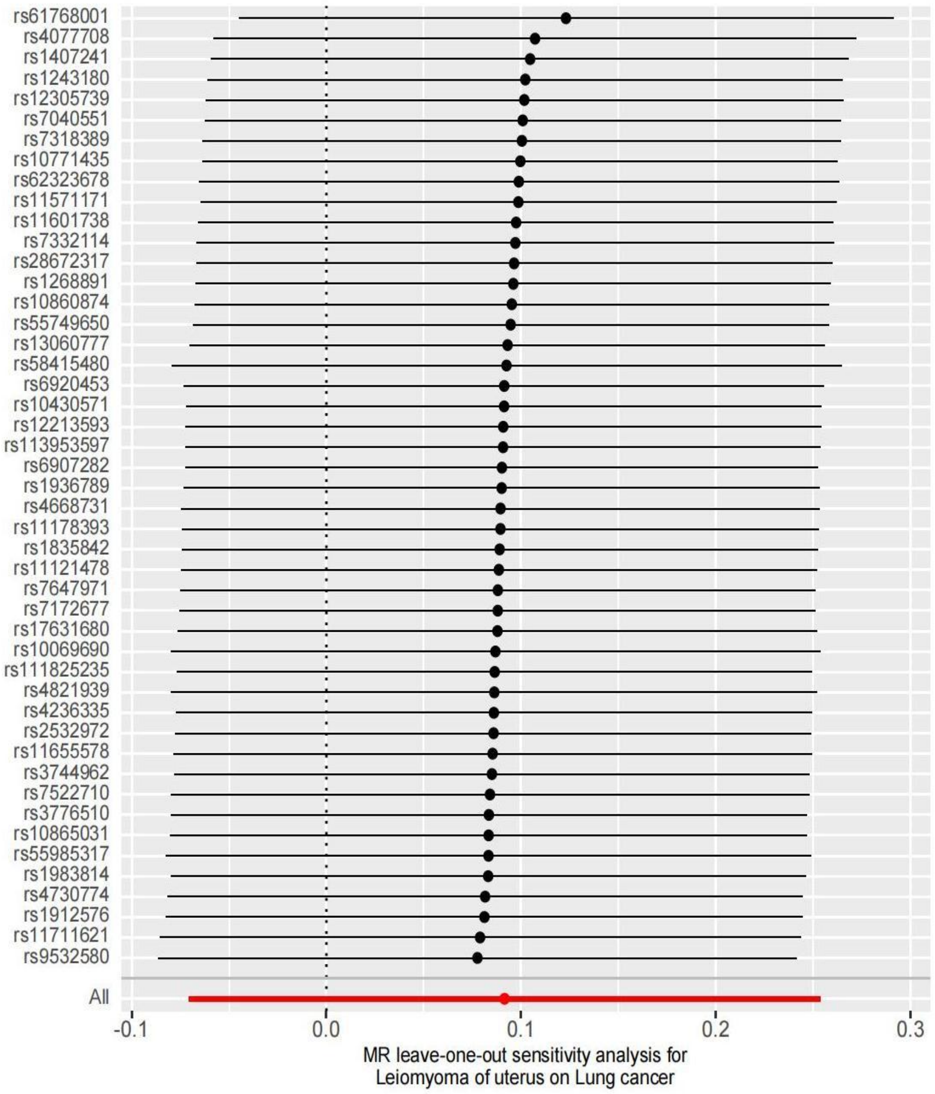

Figure S12. Leave-one-out inverse-variance weighted mendelian randomization analyses of uterine leiomyoma on lung cancer.

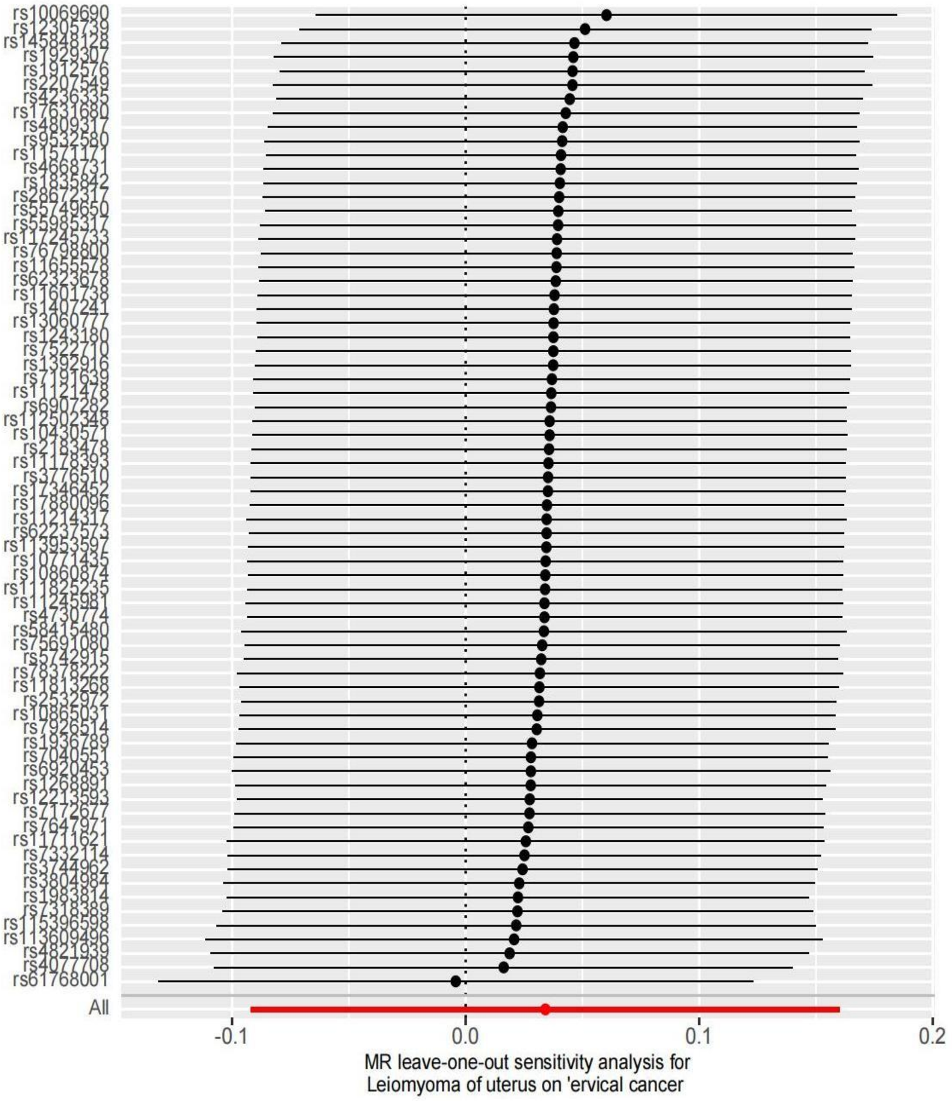

Figure S13. Leave-one-out inverse-variance weighted mendelian randomization analyses of uterine leiomyoma on cervical cancer.

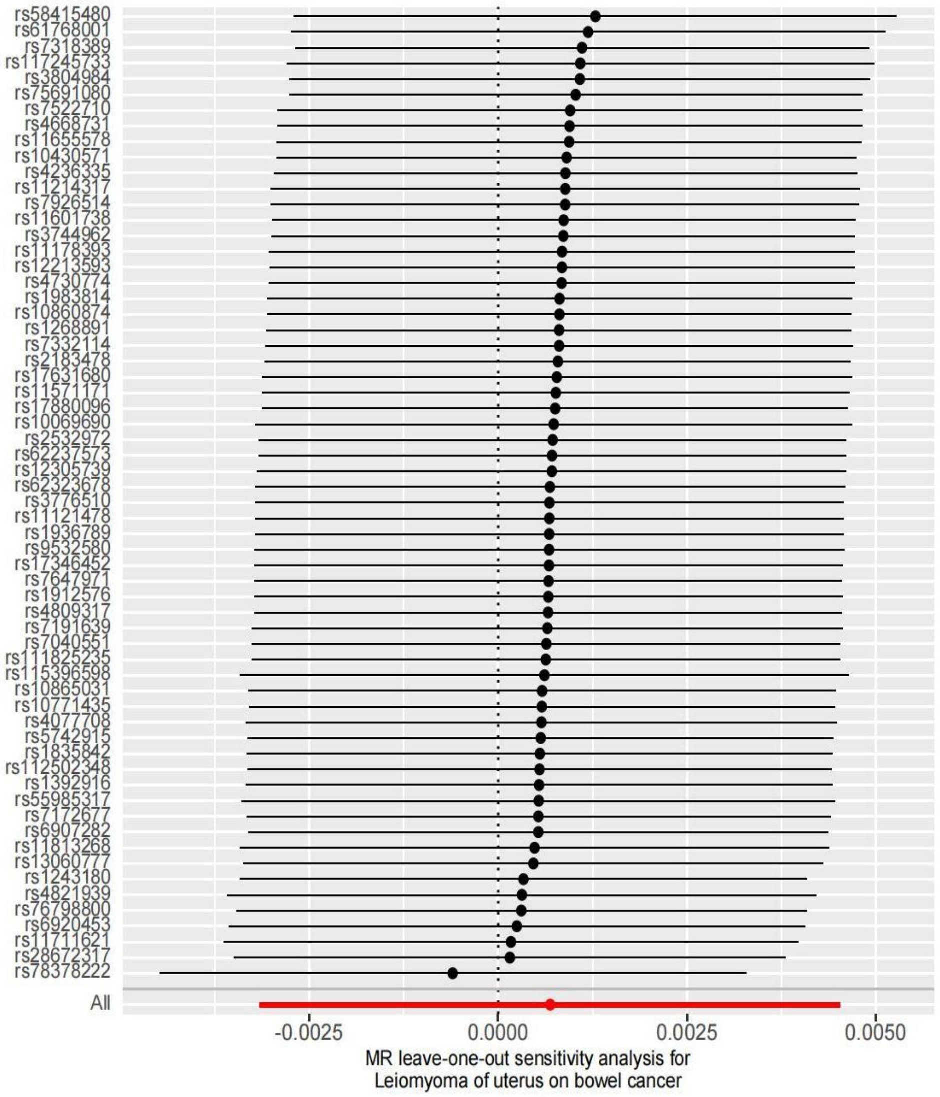

Figure S14. Leave-one-out inverse-variance weighted mendelian randomization analyses of uterine leiomyoma on bowel cancer.

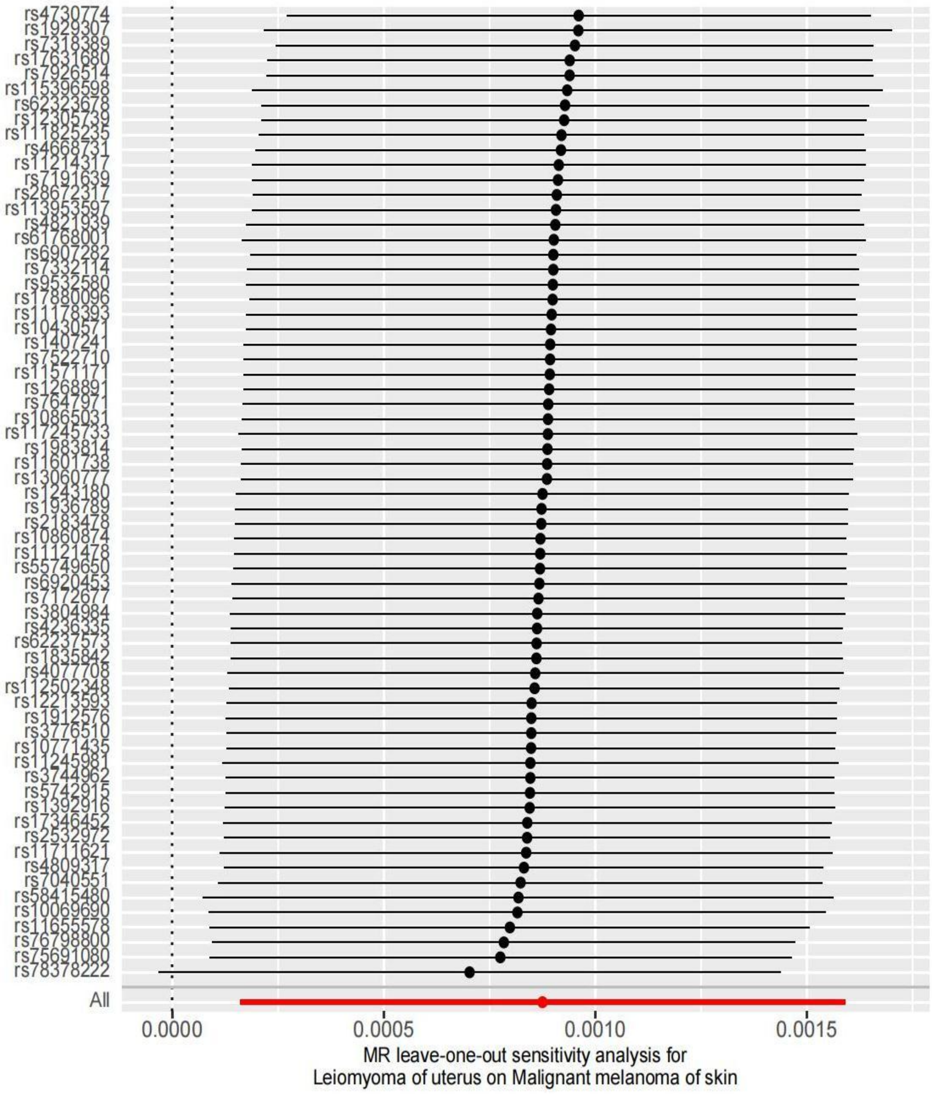

Figure S15. Leave-one-out inverse-variance weighted mendelian randomization analyses of uterine leiomyoma on malignant melanoma of skin.

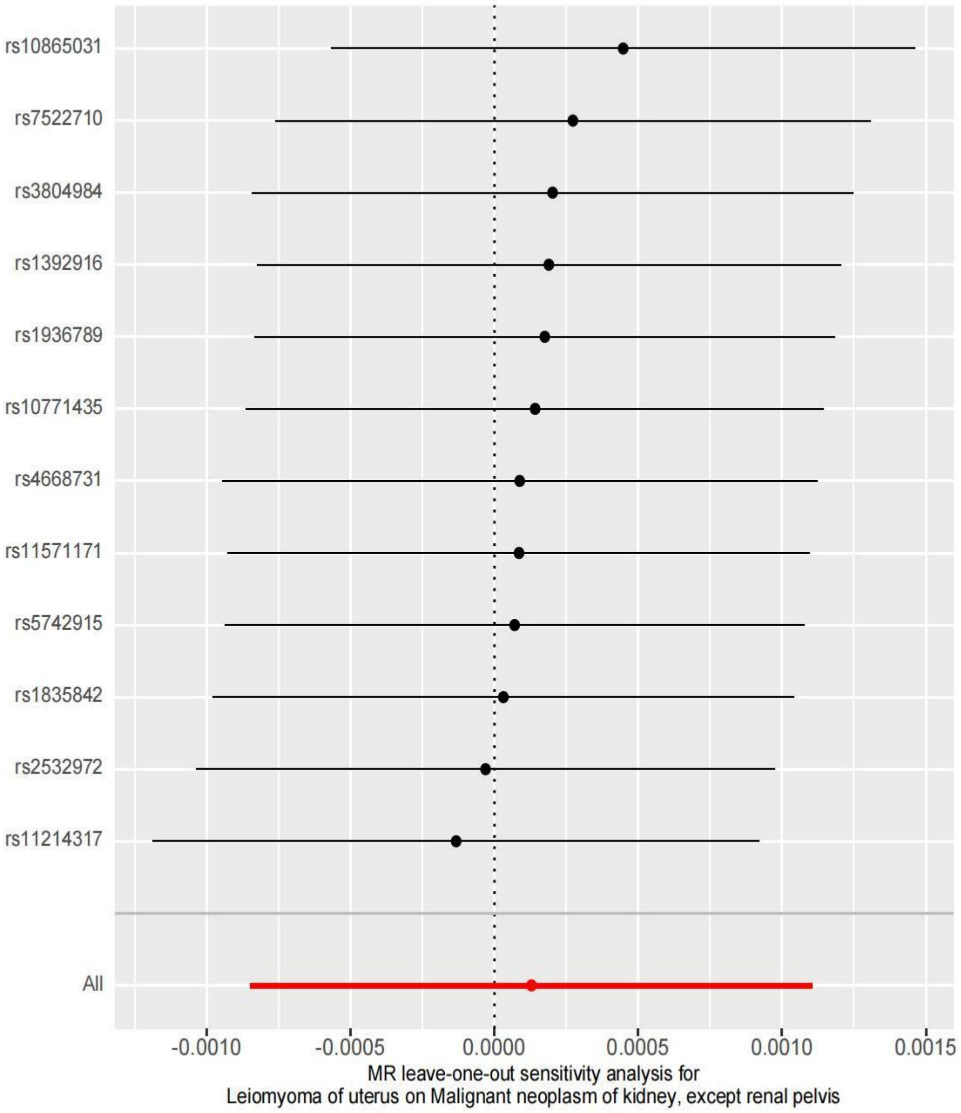

Figure S16. Leave-one-out inverse-variance weighted mendelian randomization analyses of uterine leiomyoma on malignant neoplasm of kidney.

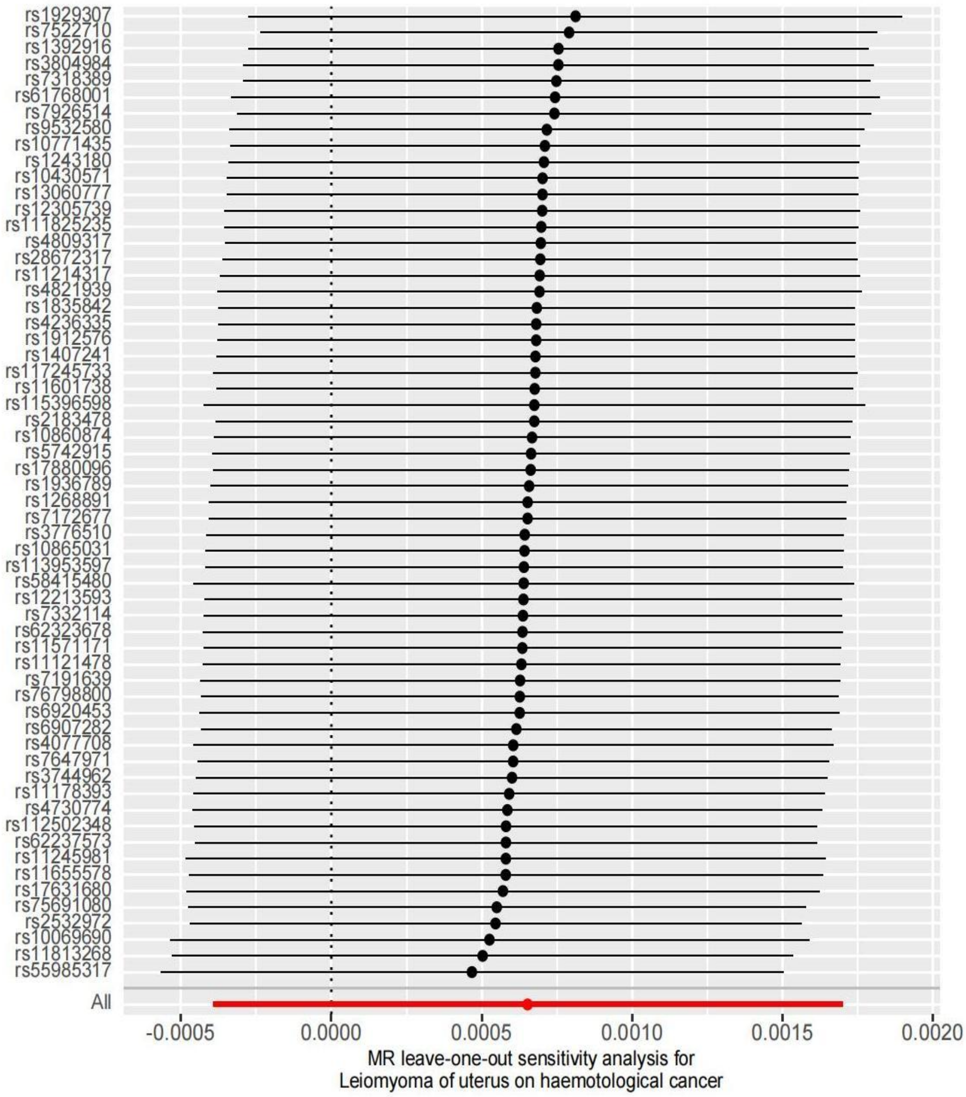

Figure S17. Leave-one-out inverse-variance weighted mendelian randomization analyses of uterine leiomyoma on haemotological cancer.

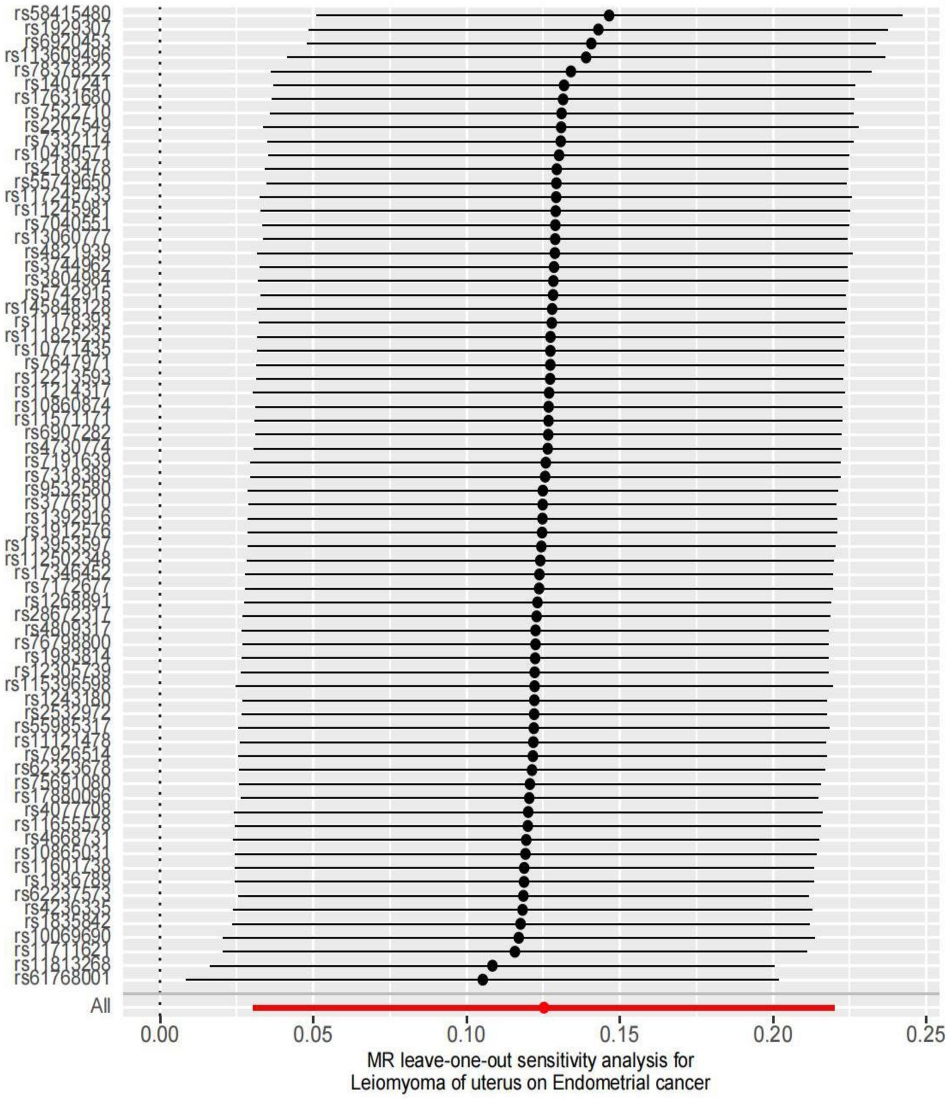

Figure S18. Leave-one-out inverse-variance weighted mendelian randomization analyses of uterine leiomyoma on endometrial cancer.

Outcome	No.of SNP	Method	OR(95% CI)	OR	P−value
Low malignant potential ovarian cancer	58	Inverse variance weighted	1.22 (1.06 to 1.40)	1.2196400	0.004
		Contamination mixture method	1.12 (0.90 to 1.39)	1.1217055	0.311
		Robust adjusted profile score (RAPS)	1.19 (1.04 to 1.37)	1.1906116	0.014
		Debiased inverse−variance weighted method	1.22 (1.07 to 1.40)	1.2237799	0.004
		Constrained maximum likelihood	1.18 (1.03 to 1.36)	1.1822260	0.018
Serous ovarian cancer	58	Inverse variance weighted	1.29 (1.10 to 1.52)	1.2916850	0.002
		Contamination mixture method	1.50 (1.07 to 2.11)	1.5033110	0.104
		Robust adjusted profile score (RAPS)	1.28 (1.07 to 1.53)	1.2800926	0.007
		Debiased inverse−variance weighted method	1.30 (1.10 to 1.53)	1.2968254	0.002
		Constrained maximum likelihood	1.25 (1.04 to 1.50)	1.2528661	0.016
Invasive mucinous ovarian cancer	61	Inverse variance weighted	1.24 (1.08 to 1.44)	1.2437419	0.003
		Contamination mixture method	1.13 (0.86 to 1.48)	1.1290516	0.241
		Robust adjusted profile score (RAPS)	1.20 (1.03 to 1.40)	1.2019550	0.016
		Debiased inverse−variance weighted method	1.25 (1.08 to 1.44)	1.2477819	0.003
		Constrained maximum likelihood	1.17 (0.98 to 1.41)	1.1747598	0.080
Clear cell ovarian cancer	58	Inverse variance weighted	1.25 (1.03 to 1.51)	1.2479690	0.023
		Contamination mixture method	1.52 (1.15 to 2.01)	1.5209844	0.011
		Robust adjusted profile score (RAPS)	1.29 (1.08 to 1.54)	1.2892976	0.005
		Debiased inverse−variance weighted method	1.25 (1.04 to 1.51)	1.2520219	0.020
		Constrained maximum likelihood	1.26 (0.99 to 1.61)	1.2618898	0.062
Breast cancer	55	Inverse variance weighted	1.07 (1.02 to 1.11)	1.0683305	0.002
		Contamination mixture method	1.10 (1.06 to 1.16)	1.1045685	0.000
		Robust adjusted profile score (RAPS)	1.08 (1.03 to 1.12)	1.0757579	0.000
		Debiased inverse−variance weighted method	1.07 (1.03 to 1.11)	1.0694939	0.001
		Constrained maximum likelihood	1.07 (1.03 to 1.11)	1.0717476	0.001
Brain tumor	69	Inverse variance weighted	1.23 (1.06 to 1.42)	1.2264991	0.007
		Contamination mixture method	1.33 (1.08 to 1.64)	1.3271879	0.027
		Robust adjusted profile score (RAPS)	1.21 (1.04 to 1.41)	1.2143854	0.012
		Debiased inverse−variance weighted method	1.23 (1.06 to 1.43)	1.2305101	0.007
		Constrained maximum likelihood	1.20 (0.99 to 1.46)	1.2003196	0.065
Gastric cancer	70	Inverse variance weighted	0.91 (0.85 to 0.98)	0.9136150	0.008
		Contamination mixture method	0.88 (0.81 to 0.95)	0.8770062	0.000
		Robust adjusted profile score (RAPS)	0.90 (0.84 to 0.97)	0.9033151	0.007
		Debiased inverse−variance weighted method	0.91 (0.85 to 0.98)	0.9123362	0.009
		Constrained maximum likelihood	0.90 (0.84 to 0.97)	0.9036977	0.008

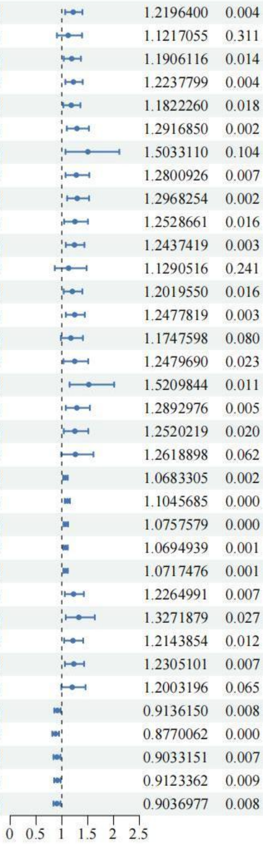

Figure S19: Forest plot of ConMix, RAPS, DIVW and CML methods to visualize the casual effect of uterine leiomyoma on seven cancers with positive results.

Outcome	No.of SNP	Method	OR(95% CI)	OR	P−value
Malignant neoplasm of corpus uterine	66	Inverse variance weighted	1.00 (1.00 to 1.00)	1.0007007	0.007
		Contamination mixture method	1.00 (1.00 to 1.00)	1.0005592	0.052
		Robust adjusted profile score (RAPS)	1.00 (1.00 to 1.00)	1.0006989	0.020
		Debiased inverse−variance weighted method	1.00 (1.00 to 1.00)	1.0007128	0.006
		Constrained maximum likelihood	1.00 (1.00 to 1.00)	1.0006901	0.026
Thyroid cancer	69	Inverse variance weighted	1.33 (1.12 to 1.56)	1.3265556	0.001
		Contamination mixture method	1.29 (1.01 to 1.65)	1.2934716	0.046
		Robust adjusted profile score (RAPS)	1.30 (1.10 to 1.55)	1.3044442	0.003
		Debiased inverse−variance weighted method	1.33 (1.13 to 1.57)	1.3323553	0.001
		Constrained maximum likelihood	1.31 (1.09 to 1.58)	1.3126910	0.003
Lung cancer	47	Inverse variance weighted	1.10 (0.93 to 1.29)	1.0957882	0.269
		Contamination mixture method	1.23 (0.93 to 1.64)	1.2349501	0.141
		Robust adjusted profile score (RAPS)	1.10 (0.93 to 1.30)	1.0968019	0.288
		Debiased inverse−variance weighted method	1.10 (0.93 to 1.29)	1.0975903	0.269
		Constrained maximum likelihood	1.09 (0.92 to 1.29)	1.0867955	0.336
Cervical cancer	70	Inverse variance weighted	1.03 (0.91 to 1.17)	1.0347078	0.596
		Contamination mixture method	1.08 (0.66 to 1.75)	1.0765464	0.555
		Robust adjusted profile score (RAPS)	1.03 (0.89 to 1.18)	1.0254146	0.726
		Debiased inverse−variance weighted method	1.04 (0.91 to 1.18)	1.0352681	0.594
		Constrained maximum likelihood	1.03 (0.90 to 1.18)	1.0302355	0.666
Bowel cancer	62	Inverse variance weighted	1.00 (1.00 to 1.00)	1.0006883	0.726
		Contamination mixture method	1.00 (1.00 to 1.01)	1.0001566	1.000
		Robust adjusted profile score (RAPS)	1.00 (1.00 to 1.00)	0.9993956	0.753
		Debiased inverse−variance weighted method	1.00 (1.00 to 1.00)	1.0006994	0.721
		Constrained maximum likelihood	1.00 (1.00 to 1.00)	1.0001433	0.943
Malignant melanoma of skin	65	Inverse variance weighted	1.00 (1.00 to 1.00)	1.0008754	0.016
		Contamination mixture method	1.00 (1.00 to 1.00)	1.0037406	1.000
		Robust adjusted profile score (RAPS)	1.00 (1.00 to 1.00)	1.0007841	0.033
		Debiased inverse−variance weighted method	1.00 (1.00 to 1.00)	1.0008904	0.015
		Constrained maximum likelihood	1.00 (1.00 to 1.00)	1.0007901	0.044
Malignant neoplasm of kidney,except renal pelvis	12	Inverse variance weighted	1.00 (1.00 to 1.00)	1.0001288	0.796
		Contamination mixture method	1.00 (1.00 to 1.00)	1.0023913	1.000
		Robust adjusted profile score (RAPS)	1.00 (1.00 to 1.00)	1.0002322	0.658
		Debiased inverse−variance weighted method	1.00 (1.00 to 1.00)	1.0001317	0.796
		Constrained maximum likelihood	1.00 (1.00 to 1.00)	1.0001333	0.801
Haemotological cancer	60	Inverse variance weighted	1.00 (1.00 to 1.00)	1.0006527	0.222
		Contamination mixture method	1.00 (1.00 to 1.00)	1.0013843	1.000
		Robust adjusted profile score (RAPS)	1.00 (1.00 to 1.00)	1.0006160	0.268
		Debiased inverse−variance weighted method	1.00 (1.00 to 1.00)	1.0006643	0.214
		Constrained maximum likelihood	1.00 (1.00 to 1.00)	1.0005179	0.316
Endometrial cancer	70	Inverse variance weighted	1.13 (1.03 to 1.25)	1.1334597	0.010
		Contamination mixture method	1.27 (0.96 to 1.68)	1.2703145	0.111
		Robust adjusted profile score (RAPS)	1.12 (1.01 to 1.25)	1.1195021	0.038
		Debiased inverse−variance weighted method	1.14 (1.03 to 1.25)	1.1356819	0.009
		Constrained maximum likelihood	1.13 (1.02 to 1.25)	1.1293697	0.019

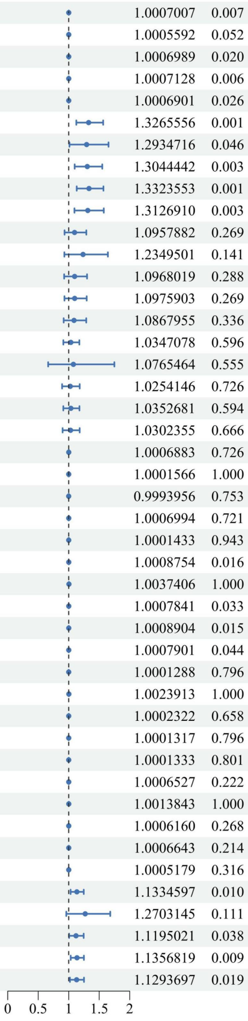

Figure S20: Forest plot of ConMix, RAPS, DIVW and CML methods to visualize the casual effect of uterine leiomyoma on six other cancers with negative results
